# Effectiveness of a Cannabinoids Supplement on Sleep and Mood in Adults With Subthreshold Insomnia: A Randomized Double‐Blind Placebo‐Controlled Crossover Pilot Trial

**DOI:** 10.1002/hsr2.70481

**Published:** 2025-02-19

**Authors:** Heather Hausenblas, Stephanie Hooper, Tarah Lynch

**Affiliations:** ^1^ Jacksonville University Jacksonville Florida USA; ^2^ University of North Florida Jacksonville North Florida USA

**Keywords:** cannabidiol, cannabinol, cannabis, randomized trial, sleep

## Abstract

**Background and Aims:**

Conduct a pilot randomized double‐blind placebo‐controlled crossover trial for adults with subthreshold insomnia symptoms to examine the effectiveness of a cannabinoids supplement on sleep quality and health outcomes.

**Methods:**

Adults with subthreshold insomnia symptoms (*N* = 20, *M*age = 47.40) were randomized to either the Cannabinoids Supplement (CS) or Placebo Condition (PC) for 10 days. The CS was an oral soft gel that contained 3 mg Δ^9^‐tetrahydrocannabinol, 6 mg cannabinol, 10 mg cannabidiol, and 90 mg of a proprietary food‐grade terpene blend. Following a 2‐week washout, they completed the alternate condition. The following validated questionnaires were collected at baseline and following each condition: Insomnia Severity Index, Pittsburgh Sleep Quality Index, Bergen Insomnia Scale, Profile of Mood States (POMS), Perceived Stress Scale, Pain and Sleep Questionnaire. Trait Anxiety Inventory, Flinders Daytime Fatigue, and Health‐related Quality of Life Scale. Clinical trial registry number = ISRCTN 15022302.

**Results:**

When compared to PC, the CS Condition had significantly improved sleep quality/efficiency, insomnia symptoms, and health‐related quality of life, *p* < 0.05. Nonsignificant improvements for the CS compared to the PC were found for the POMS mood subscales of tension, anger, fatigue, depression, and vigor, as well as anxiety. The Esteem subscale improved significantly from Baseline to Post for the PC. Both the CS and PC Vigor improved significantly from baseline. Anxiety improved significantly from Baseline to Post for the CS. No adverse events reported.

**Conclusion:**

This cannabinoid‐based formulation was a well‐tolerated oral supplement that may improve adults' sleep quality/efficiency and health‐related quality of life. Larger controlled trials are encouraged to examine the longer‐term effects of this supplement in a variety of populations and environments.

## 1 Introduction

Sleep is essential for maintaining physical, social, and psychological well‐being. For example, sleep is a fundamental pillar of mental health, playing a crucial role in emotional regulation, cognitive function, and overall well‐being [1, 2]. Adequate and restorative sleep not only supports daily functioning but is also integral in the body's ability to manage stress effectively. Stress, a common trigger for psychological and physiological disorders, can disrupt sleep patterns, creating a cycle that exacerbates mental health challenges. Particularly in individuals with autoimmune diseases, sleep disturbances are prevalent and linked to heightened inflammatory responses, worsened disease activity, and an increased risk of psychosomatic symptoms [3].

Chronic stress can exacerbate autoimmune conditions by dysregulating the hypothalamic‐pituitary‐adrenal axis and promoting systemic inflammation. Similarly, poor sleep quality has been associated with the onset and progression of psychosomatic symptoms, such as fatigue, pain, and gastrointestinal discomfort, which are common in stress‐related disorders. This highlights the importance of interventions targeting sleep health as a means to combat stress and improve overall quality of life [1, 2, 3].

Despite the well‐established importance of sleep, dissatisfaction remains prevalent among the general population. Up to 41.7% of adults report insufficient rest, and 48% experience difficulties with sleep initiation or maintenance [4]. Although medication can be effective for treating sleep disorders, there is an unmet need for safe and accessible sleep aids for adults with nonclinical sleep issues [5, 6].

Traditional interventions involving over‐the‐counter and prescription medications often come with undesirable side effects, limited efficacy, and the potential for dependency [7]. Consequently, there is a need for research into alternative interventions to enhance sleep quality and daytime functioning in individuals with poor sleep habits. Investigating nonpharmacological approaches could provide safer and more effective solutions for improving overall sleep health.

Despite the extensive use of herbal plants to address various health concerns, randomized controlled trials evaluating their effectiveness in enhancing sleep quality remain limited. Among herbal remedies, cannabinoid compounds derived from the cannabis plant have gained significant attention as potential alternative treatments for a variety of conditions, including sleep disturbances. Notably, compounds such as cannabidiol (CBD), cannabinol (CBN), and tetrahydrocannabinol (THC) are increasingly used to address poor sleep quality and symptoms of insomnia. Emerging research provides preliminary evidence supporting these sleep‐related claims, highlighting the need for further rigorous investigation [8, 9, 10, 11, 12, 13, 14, 15, 16].

Recommendations indicate that further randomized controlled trials are essential to substantiate the sleep claims of cannabis products containing CBN, THC, and CBN. These trials should utilize validated self‐report measures and investigate dosages higher than those typically found in currently available cannabis products marketed for sleep (typically ≤ 5 mg for CBN). Additionally, rigorous scientific evaluation using randomized controlled designs of these products can provide a clearer understanding of their efficacy and safety, ensuring that consumers receive reliable information based on solid evidence [17]. However, before large‐scale studies are undertaken, pilot studies are essential to determine efficacy.

Thus, the study purpose was to address these shortcomings and conduct a pilot randomized double‐blind placebo‐controlled crossover trial for adults with subthreshold insomnia symptoms to examine the effectiveness of a cannabinoid supplement containing CBD and CBN on sleep quality and mood. Subthreshold insomnia, also known as mild or subclinical insomnia, refers to sleep disturbances that do not meet the full criteria for clinical insomnia but still impact sleep quality and daily functioning. The prevalence of subthreshold insomnia varies across studies, with estimates ranging from 27% to 32% in the general adult population [1, 2, 3]. The primary outcome was sleep quality assessed with validated self‐report measures. The secondary outcomes were health‐related quality of life, anxiety, perceived stress, mood, daytime fatigue, and safety/adverse events.

## 2 Methods

### 2.1 Participants

Participants were 20 adults (*M*age = 47.40) with subthreshold insomnia symptoms (as determined by the Insomnia Severity Index (ISI), *M* = 13.15, SD = 4.50 [18].

### 2.2 Exclusion Criteria

Participants were excluded if they had (1) severe insomnia based on the Insomnia Severity Index (ISI ≥ 22 for clinical insomnia and ISI < 8 for nonclinical insomnia); (2) history of a diagnosed disorder affecting sleep quality; (3) reported events that could cause severe stress within 2 weeks of baseline; (4) use of medication that could influence sleep patterns within 1 month of the trial; (5) use of hormone therapy; (6) binge alcohol consumption; (7) smoking; (8) high caffeine intake; (9) work schedule that causes irregular sleep patterns; (10) history of travel to a different time zone within 1 month of study; (11) low or high body mass index (BMI ≤ 18 kg/m^2^ or ≥ 35 kg/m^2^); (12) pregnant, trying to conceive, or breastfeeding; (13) taking sleep supplements or medication, (14) unwilling to abstain from other cannabis/hemp product use for 2 weeks before and during the trial, and (15) individuals deemed unable to complete the protocol as designed.

### 2.3 Intervention

Using a randomized double‐blind placebo‐controlled crossover trial, the participants were randomized to either the cannabinoid supplement (CS) or placebo control (PC) condition for 10 days. All participants signed an Institutional Approved Informed Consent (Sterling IRB). Following a 2‐week washout period, the participants completed the alternate condition for 10 days. The CS was an oral soft gel that contains 3 mg THC, 6 mg CBN, 10 mg CBD, and 90 mg of a proprietary food‐grade terpene blend (https://sannasleep.com/). The placebo oral soft gel contained medium chain trigyceride (MCT) oil. The supplement was taken an hour before nighttime sleep. Adherence was 100%. The data were collected from January 1, 2022 to December 12, 2022.

### 2.4 Trial Reporting

The trial was conducted and reported in accordance the Consolidated Standards of Reporting Trials (including reporting of harms and the guidelines for reporting of statistics for clinical research in urology to ensure transparency and completeness [19, 20].

### 2.5 Study Design

This study was approval by Sterling Institutional Review Board (10333) in compliance with ethical principles regarding human participant research and registered with ISRCTN clinical trial registry (ISRCTN 15022302).

This study involved a double‐blind, placebo‐controlled, randomized, crossover design in which participants cycled through two independent treatment arms. Both the participants and research team were blinded to the conditions (i.e., double blinded). The participants were randomized to both the Intervention (A) and Placebo (B) arms. Participants were randomized to the two conditions (Intervention A and Placebo B) using a computer‐generated randomization sequence to ensure allocation concealment. This process ensured that each participant had an equal chance of starting in either treatment arm, thereby minimizing potential biases and balancing confounding variables across the study conditions. The study had a 1‐week run‐in/baseline period during which participants were monitored for protocol compliance to ensure data was collected correctly. The run‐in/baseline period was followed by two independent 10‐day treatment periods (A/B and B/A), which were each be followed by a 1‐week wash‐out period, which is consistent with previous cannabis crossover design research [21, 22]. A 1‐week wash‐out period was chosen to rule out any carry‐over effects, as the elimination half‐life of CBD is two‐5 days after chronic oral administration [21, 22].

The independent variable was the Sanna dietary supplementation. The dependent variables were sleep quality (primary outcome) and mood, anxiety, stress, and health‐related quality of life (secondary outcomes).

Power analysis: Crossover designs reduce variability by accounting for within‐subject differences, enhancing statistical power. Paired‐samples t‐test were used for analysis since participants act as their own control in a crossover design. Prior research shows a moderate effect between Experimental and Placebo conditions in cannabis supplementation and sleep intervention studies (Narayan et al., 2024; Ried et al., 2023; Wang et al., 2024) [21, 22, 23].

For a randomized double‐blind placebo‐controlled crossover study with two conditions, expecting a moderate effect size (Cohen's *d* = 0.5) and aiming for 80% power with a significance level of 0.05, approximately 20 participants are needed. Each participant will experience both conditions due to the crossover design, which improves the efficiency of the study.

### 2.6 Blinding

Participants were automatically assigned to their respective groups by a password protected computer program upon enrollment. This ensured complete allocation concealment by preventing researchers and participants from knowing which group they would be assigned to before enrollment, thereby minimizing selection bias. A blinded research assistant generated the random allocation sequency, enrolled participants, and assigned participants to the intervention. To ensure that all participants and researchers were unaware of the treatment assignments, Sanna Inc., provided the supplement/placebo labelled as either A or B. The supplement/placebo pills were identical in color, odor, and size. At the conclusion of the study, immediately following the last assessment, the research team was unblinded. The participants were then unblinded and informed of their assigned condition.

### 2.7 Adherence

**Figure 1:**
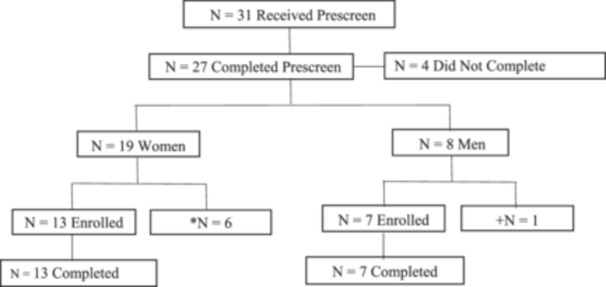
Participant flow chart. *N = 6 excluded because they did not meet the inclusion criteria. +*N* = 1 excluded because failed to meet inclusion criteria.

Of the 27 people who completed the prescreen 7 were excluded from participating for the following reasons: *n* = 1 stopped responding, *n* = 1 had personal reasons before the start of the study, *n* = 2 had diagnosed heart conditions, *n* = 2 scored too low on ISI, and *n* = 1 had issues with receiving supplement and ring at home address. Of the 20 participants who enrolled and provided consent, all 20 completed the trial, resulting in a completion rate of 100% (see Table 1 and Figure 1).

**Table 1:** Sleep quality outcomes of the Insomnia Severity Index, Pittsburg Sleep Quality Index (PSQI), Bergen Insomnia Scale, Restorative Sleep Scale, Flinder's Fatigue Scale, and Pain and Sleep Questionnaire Baseline and post conditions mean, standard deviation, and paired sample *t*‐test results (df = 19) on delta scores and prepost scores by condition.

Item	Baseline *M* (SD)	Postsleep supplement *M* (SD)	*t*‐test on sleep supplement (baseline vs. post)	95% confidence interval (CI)	Post Placebo *M* (SD)	*t*‐test on placebo (baseline vs. post)	95% confidence interval (CI)	*t*‐test on delta scores	95% confidence interval (CI)
Insomnia Severity Index	12.30 (3.99)	8.55 (5.32)	*t* = 3.47, *p* = 0.003**	1.49 to 6.01	9.00 (4.97)	*t* = 4.03, *p* = 0.001^+^	1.59 to 5.01	*t* = −0.39, *p* = 0.70	−2.85 to 1.95
PSQI: Sleep Quality	1.60 (0.68)	1.05 (0.83)	*t* = 2.6, *p* = 0.02**	0.1 to 0.92	1.30 (0.66)	*t* = 1.39, *p* = 0.18	−0.08 to 0.4	*t* = −1.78, *p* = 0.05*	−0.76 to 0.06
PSQI: Sleep Latency	0.60 (0.75)	0.40 (0.75)	*t* = 3.44, *p* = 0.003**	0.17 to 0.71	0.30 (0.66)	*t* = 3.75, *p* = 0.001^+^	0.21 to 0.73	*t* = 0.58, *p* = 0.57	−0.08 to 0.14
PSQI: Sleep Duration	1.05 (0.76)	0.85 (0.67)	*t* = 1.07, *p* = 0.30	−0.19 to 0.59	0.95 (0.83)	*t* = 1.08, *p* = 0.29	−0.19 to 0.59	*t* = 0.01, *p* = 0.99	−0.43 to 0.43
PSQI: Sleep Efficiency	0.45 (0.69)	0.40 (0.82)	*t* = 0.16, *p* = 0.87	−0.3 to 0.35	0.70 (0.92)	*t* = −2.22, *p* = 0.04^+^	−0.56 to −0.02	*t* = −2.04, *p* = 0.05*	−0.64 to 0.01
PSQI: Sleep Disturbances	1.25 (0.44)	1.2 (0.52)	*t* = −0.1, *p* = 0.92	−0.23 to 0.21	1.15 (0.49)	*t* = 0.48, *p* = 0.64	−0.14 to 0.23	*t* = 0.59, *p* = 0.56	−0.13 to 0.24
PSQI: Use of Sleep Medication	0.55 (1.00)	0.50 (0.95)	*t* = 0.4, *p* = 0.69	−0.23 to 0.34	0.15 (0.49)	*t* = 2.74, *p* = 0.01^+^	0.06 to 0.44	*t* = 2.31, *p* = 0.03*	0.02 to 0.37
PSQI: Daytime Dysfunction	0.85 (0.67)	0.75 (0.72)	*t* = 0.57, *p* = 0.58	−0.27 to 0.47	0.9 (0.72)	*t* = −0.57, *p* = 0.58	−0.23 to 0.13	*t* = −0.9, *p* = 0.38	−0.5 to 0.2
PSQI: Global Sleep Quality	6.12 (2)	4.86 (2.14)	*t* = 2.75, *p* = 0.01**	0.3 to 2.22	5.45 (1.72)	*t* = 1.83, *p* = 0.08	−0.1 to 1.44	*t* = −1.02, *p* = 0.32	−1.79 to 0.62
Bergen Insomnia Scale	21.40 (10.25)	12.70 (8.43)	*t* = 3.6, *p* = 0.002**	3.65 to 13.76	16.55 (9.44)	*t* = 2.33, *p* = 0.03^+^	0.5 to 9.21	*t* = −1.76, *p* = 0.95	−8.43 to 0.73
Restorative Sleep Scale	24.30 (4.18)	25.45 (3.49)	*t* = −1.24, *p* = 0.23	−3.08 to 0.78	25.80 (3.65)	*t* = −1.87, *p* = 0.08	−3.18 to 0.18	*t* = −0.35, *p* = 0.73	−2.42 to 1.72
Flinder's Fatigue Scale	10.93 (5.07)	8.61 (4.64)	*t* = 2.26, *p* = 0.04**	0.18 to 4.46	9.24 (5.00)	*t* = 1.37, *p* = 0.19	−0.88 to 4.24	*t* = −0.54, *p* = 0.59	−3.08 to 1.81
Pain and Sleep: Awakened by Pain During Night	1.82 (3.34)	3.48 (5.94)	*t* = −1.65, *p* = 0.12	−7.46 to 0.89	2.21 (4.42)	*t* (19) = −1.18, *p* = 0.25	−3.52 to 0.98	*t* = 0.75, *p* = 0.46	−1.64 to 5.67
Pain and Sleep: Trouble Falling Asleep	1.32 (3.12)	4.60 (8.34)	*t* = −1.21, *p* = 0.24	−4.53 to 1.21	2.59 (4.07)	*t* = −0.3, *p* = 0.77	−3.08 to 2.3	*t* = 1.15, *p* = 0.26	−2.26 to 4.8
Pain and Sleep: Awakened by Pain in Morning	1.22 (3.00)	3.22 (6.06)	*t* = −1.39, *p* = 0.18	−5.02 to 1.02	1.48 (3.84)	*t* = −0.28, *p* = 0.79	−2.23 to 1.71	*t* = 1.18, *p* = 0.25	−1.35 to 4.83

^+^ Significant baseline versus post PC.

* Significant condition difference based on delta scores [(Baseline–Post SS) vs. (Baseline–Post PC)].

** Significant baseline versus post SS condition.

### 2.8 Measures

The following psychometrically validated self‐report measures were completed at Baseline, and following each condition:

### 2.9 Insomnia Severity Index

The Insomnia Severity Index is a 7‐item self‐report measure assessing symptoms of poor sleep. This index assesses sleep onset, sleep maintenance, early morning awakening problems; sleep dissatisfaction; interference of sleep difficulties with daytime functioning; whether sleep problems are noticed by others; and distress caused by sleep difficulties. The Insomnia Severity Index has excellent internal consistency (Cronbach *α* = 0.91) [18].

### 2.10 Pittsburgh Sleep Quality Index (PSQI)

The PSQI is a standardized, self‐administered questionnaire that evaluates retrospective sleep quality and disturbances [12]. The PSQI consists of 19 items forming the following seven subscales: (1) sleep quality, (2) sleep latency, (3) sleep duration, (4) sleep efficiency, (5) sleep disturbance, (6) sleep medication, and (7) daily dysfunction. Each item has a score that ranges from 0 to 3. The scores of seven components are summed to yield a global score ranging from 0 to 21. Higher scores indicate poorer sleep quality [24].

### 2.11 Bergen Insomnia Scale

The Bergen Insomnia scale measures insomnia in accordance with the DSM‐IV criteria for insomnia. The scale consists of six items, where the first four items assess difficulties with sleep initiation, sleep maintenance, early morning awakening, and nonrestorative sleep. The last two items assess daytime impairments and satisfaction with sleep. Each item is given a value of 0–7, ranging from the number of days on average the person has had any troubles with the listed symptoms over the past month. The scale has a max score of 42, and a minimum of 0.2 [5].

### 2.12 Restorative Sleep Questionnaire

The Restorative Sleep Questionnaire is a validated 11‐item questionnaire that assesses restorative sleep by asking respondents to rate on a 5‐point scale feelings of tiredness, mood, and energy [13]. The Restorative Sleep Questionnaire has good psychometric properties and is able to distinguish between healthy controls, patients with primary insomnia, and insomnia patients with isolated nonrestorative sleep complaints [25].

### 2.13 Profile of Mood States (POMS) Questionnaire

The POMS‐40 was used to assess the mood states of tension, anger, vigor, fatigue, depression, and confusion [26]. A composite score was computed by summing each of the individual scores for tension, depression, anxiety, fatigue, and confusion, with vigor scores subtracted to indicate patients' total mood disturbance. Each item of the POMS items was scored on a 5‐point Likert scale ranging from 0 (not at all) to 4 (extremely) with lower scores indicated an improved mood. This scale has good to excellent reliability and validity.

### 2.14 Flinders Fatigue Scale

The Finders Fatigue Scale is a 7‐item scale that measures various characteristics of fatigue (e.g., frequency, severity) experienced over the past 2 weeks [15]. The items tap into commonly reported themes of how problematic fatigue is, the consequences of fatigue, frequency, severity, and insomnia patients' perception of fatigue's association with sleep. Six items are presented in Likert format, with responses ranging from 0 (not at all) to 4 (extremely). Item 5 measures the time of day when fatigue is experienced and uses a multiple‐item checklist. Respondents can indicate more than one response for item 5, and it is scored as the sum of all times of the day indicated by the respondent. One item explicitly asks for respondents' impression of whether they attribute their fatigue to their sleep. Total fatigue is calculated as the sum of all individual items. Total fatigue scores range from 0 to 31, with higher scores indicating greater fatigue. A clear description of the term “fatigue” is provided in the initial instructions to the scale [27].

### 2.15 Perceived Stress Scale

The Perceived Stress Scale‐4 measures the degree to which individuals appraise situations in their lives as stressful [16]. Specifically, the scale evaluates the degree to which individuals believe their life has been unpredictable, uncontrollable, and overloaded during the previous 2 weeks. The items are general in nature rather than focusing on specific events or experiences. The scale consisted of four items, and each item was scored on a 5‐point Likert scale ranging from 0 (“never”) to 4 (“very often”) with higher scores indicating more perceived stress. This scale has excellent psychometric properties [28].

### 2.16 Pain and Sleep Questionnaire‐3

The three‐item Pain and Sleep Questionnaire measures the impact of pain on quality of sleep and includes the following three items: “1. How often have you had trouble falling asleep because of pain?”, “2. How often have you been awakened by pain during the night?”, and “3. How often have you been awakened by pain in the morning?” [18]. The possible answers range from 0 indicating “never” to 100 representing “always”. This questionnaire has excellent psychometric properties [29].

### 2.17 Trait Anxiety Inventory

The Trait Anxiety Inventory (20‐items) was used to measure general feelings of anxiety including general states of calmness, confidence, and security.20 Higher scores indicate more severe anxiety levels. Each item was assessed on a 4‐point Likert‐type scale (from 0 to 3 points) [30].

### 2.18 The CDC Health‐Related Quality of Life (HRQoL)

The HRQoL measure was used to assess participants' quality of life related to their health. This tool includes four core questions evaluating self‐perceived health status, the number of recent days of poor physical and mental health, and activity limitations due to health problems. The HRQoL measure is well‐regarded for its excellent psychometric properties, including high reliability and validity, making it a robust tool for evaluating health‐related quality of life in research and clinical settings [31].

### 2.19 Statistical Analysis

Data were assessed for normality by evaluating skewness and kurtosis values, conducting the Shapiro‐Wilk test, and inspecting Q‐Q plots. Outliers were defined as data points exceeding three interquartile ranges beyond the 25% and 75%. No extreme outliers were identified. For the primary analysis, paired‐samples *t*‐tests were employed, as participants served as their own controls in the crossover design. A two‐sided significance level (*p* < 0.05) was used to evaluate outcomes in line with a priori significance criteria. The analyses were performed using Microsoft Excel and IBM Statistical Package for the Social Sciences (version 29).

## 3 Results

For sleep outcomes, the CS condition had significant improvements in the PSQI measures of sleep quality and sleep efficiency compared to the PC condition (see Table 1). The CS condition also had significant improvements in baseline for sleep latency, sleep duration, and global sleep quality. Larger improvements in the Bergen Insomnia Scale and Insomnia Severity Index for the CS condition compared to the PC were also evidenced, albeit nonsignificant. Significant improvements from Baseline to Post CS were evidenced for the Flinders Fatigue Scale. The pain affecting sleep items were in the direction favoring the CS compared to placebo, albeit nonsignificant.

For HRQoL, significant improvements in “mental health,” “feeling healthy and full of energy,” and “poor physical or mental health prevented doing usual activities” (e.g., self‐care, work, or recreation) were found for the CS condition compared to the PC (see Table 2). The CS condition had significantly less daytime fatigue than baseline (*p* < 0.05), but the improvement compared to the PC was nonsignificance (*p* = 0.19). Improvement trends in the CS condition compared to the PC were also found for the POMS. Specifically, the Anger and Fatigue subscales improved significantly from baseline for the CS condition. The Esteem subscale improved significantly from Baseline to Post for the PC. Both the CS and PC Vigor improved significantly from baseline. Anxiety improved significantly from Baseline to Post for the CS (see Table 3).

**Table 2:** Health‐related quality of life mean (*M*), standard deviation (SD), and paired sample *t*‐test results (df = 19) on delta scores and prepost scores by condition.

Item	Baseline *M* (SD)	Postsleep supplement *M* (SD)	*t*‐test on sleep supplement (baseline vs. post)	95% confidence interval (CI)	Post Placebo *M* (SD)	*t*‐test on Placebo (baseline vs. post)	95% confidence interval (CI)	*t*‐test on delta scores	95% confidence interval (CI)
Overall quality of life	3.84 (0.67)	4.31 (0.65)	*t* = −3.15, *p* = 0.01**	−0.79 to −0.16	4.15 (0.67)	*t* = −2.57, *p* = 0.02^+^	−0.57 to −0.06	*t* = 1.22, *p* = 0.24	−0.11 to 0.43
Physical health not good	2.85 (3.72)	0.81 (1.06)	*t* = 2.55, *p* = 0.02**	0.36 to 3.71	1.15 (1.31)	*t* = 2.11, *p* = 0.05^+^	0.02 to 3.39	*t* = −1.00, *p* = 0.33	−1.03 to 0.36
Mental health not good	1.47 (1.51)	0.99 (1.11)	*t* = 1.96, *p* = 0.07	−0.03 to 0.99	1.76 (2.34)	*t* = −0.77, *p* = 0.45	−1.08 to 0.5	*t* = −2.23, *p* = 0.04*	−1.5 to −0.05
Poor health prevented activity	2.20 (3.09)	0.48 (0.75)	*t* = 2.69, *p* = 0.01**	0.38 to 3.06	1.08 (1.36)	*t* = 1.89, *p* = 0.07	−0.12 to 2.37	*t* = −2.02, *p* = 0.05*	−1.21 to 0.02
Pain made activities hard	0.68 (1.05)	0.60 (0.82)	*t* = 0.33, *p* = 0.75	−0.45 to 0.61	0.81 (0.96)	*t* = −0.45, *p* = 0.66	−0.75 to − 0.48	*t* = −0.84, *p* = 0.41	−0.75 to 0.32
Felt sad, blue, depressed	0.92 (1.41)	0.72 (0.93)	*t* = 0.64, *p* = 0.53	−0.46 to 0.87	1.13 (1.89)	*t* = −0.89, *p* = 0.38	−0.7 to 0.28	*t* = −1.08, *p* = 0.29	−1.21 to 0.38
Felt worried, tense, anxious	2.58 (2.36)	3.00 (3.06)	*t* = −0.81, *p* = 0.43	−1.52 to 0.67	2.03 (1.89)	*t* = 1.94, *p* = 0.07	−0.04 to 1.14	*t* = 1.68, *p* = 0.11	−0.24 to 2.19
Felt did not sleep enough	5.75 (3.06)	4.35 (3.53)	*t* = 1.69, *p* = 0.11	−0.33 to 3.13	3.90 (2.73)	*t* = 3.28, *p* = 0.004^+^	0.67 to 3.03	*t* = 0.55, *p* = 0.59	−1.27 to 2.17
Felt healthy/full of energy	4.35 (2.74)	6.35 (2.98)	*t* = −3.30, *p* = 0.004**	−3.27 to −0.73	4.95 (2.93)	*t* = −1.33, *p* = 0.20	−1.54 to 0.34	*t* = 1.77, *p* = 0.05*	−0.25 to 3.05

^+^ Significant baseline versus post PC.

* Significant condition difference based on delta scores [(Baseline–Post SS) vs. (Baseline – Post PC)].

** Significant Baseline versus post SS condition.

**Table 3:** Profile of mood states (POMS), anxiety, and perceived stress baseline and postconditions mean (*M*), standard deviation (SD), and paired sample *t*‐test results (df = 19) on delta scores and prepost scores by condition.

Item	Baseline *M* (SD)	Post Sleep supplement *M* (SD)	95% confidence interval (CI)	*t*‐test on sleep supplement (baseline vs. post)	Post Placebo *M* (SD)	*t*‐test on Placebo (baseline vs. post)	95% confidence interval (CI)	*t*‐test on delta scores	95% confidence interval (CI)
POMS Tension	7.05 (5.73)	5.40 (4.68)	−0.33 to 3.63	*t* = 1.74, *p* = 0.10	6.41 (5.11)	*t* = 0.67, *p* = 0.51	−1.38 to 2.67	*t* = −1.16, *p* = 0.26	−2.82 to 0.81
POMS Anger	4.25 (3.09)	2.82 (2.33)	0.46 to 2.39	*t* = 3.08, *p* = 0.01**	3.37 (2.70)	*t* = 1.74, *p* = 0.10	−0.18 to 1.94	*t* = −1.29, *p* = 0.21	−1.42 to 0.34
POMS Fatigue	7.24 (3.58)	5.24 (3.37)	0.36 to 3.64	*t* = 2.55, *p* = 0.02**	6.24 (3.32)	*t* = 1.27, *p* = 0.22	−0.65 to 2.65	*t* = −1.38, *p* = 0.18	−2.52 to 0.52
POMS Depression	2.64 (2.69)	2.03 (1.75)	−0.43 to 1.65	*t* = 1.22, *p* = 0.24	2.68 (3.38)	*t* = −0.11, *p* = 0.91	−0.94 to 0.84	*t* =− 0.97, *p* = 0.34	−2.07 to 0.76
POMS Esteem	16.72 (3.06)	17.45 (3.50)	−1.67 to 0.21	*t* = −1.63, *p* = 0.12	18.04 (3.06)	*t* = −2.14, *p* = 0.05^+^	−2.6 to −0.03	*t* = −0.92, *p* = 0.37	−1.92 to 0.75
POMS Vigor	9.08 (3.53)	11.95 (4.38)	−4.4 to −1.35	*t* = −3.94, *p* = 0.001*	11.61 (3.79)	*t* = −3.91, *p* = 0.001^+^	−3.88 to −1.18	t = 0.4, *p* = 0.69	−1.44 to 2.13
POMS Confusion	4.71 (3.39)	3.44 (2.60)	0.41 to 2.13	*t* = 3.11, *p* = 0.01	3.71 (3.16)	*t* = 2.28, *p* = 0.03^+^	0.08 to 1.93	t = −0.54, *p* = 0.60	−1.29 to 0.76
POMS Total	152.03 (14.00)	149.00 (11.53)	−1 to 7.06	*t* = 1.57, *p* = 0.13	150.94 (10.94)	*t* = 0.51, *p* = 0.62	−3.44 to 5.63	*t* = −1.39, *p* = 0.18	−4.84 to 0.98
Anxiety Inventory	43.83 (12.30)	38.00 (11.16)	2.08 to 9.58	*t* = 3.26, *p* = 0.004*	40.96 (11.29)	*t* = 1.47, *p* = 0.16	−1.22 to 6.97	*t* = −1.30, *p* = 0.21	−7.71 to 1.79
Perceived Stress Scale	4.39 (2.85)	3.90 (3.08)	−0.46 to 1.44	*t* = 1.08, *p* = 0.29	3.66 (2.68)	*t* = 1.2, *p* = 0.24	−0.54 to 2.01	*t* = 0.36, *p* = 0.72	−1.17 to 1.66

^+^ Significant Baseline versus Post PC.

* Significant condition difference based on delta scores [(Baseline–Post SS) vs. (Baseline–Post PC)].

** Significant Baseline versus post SS condition.

Supplement adherence was 98%, and no adverse events were reported.

## 4 Discussion

The purpose was to examine the effectiveness of a hemp‐based supplement with CBD, CBN, THC, and a standardized terpene blend on sleep quality/quantity, anxiety, perceived stress, mood, pain, and HRQoL in adults with subthreshold insomnia symptoms using a randomized double‐blind placebo‐controlled crossover pilot trial. We found the CS was well‐tolerated and resulted in significant improvements in several sleep quality and health outcomes compared to both baseline and placebo.

Specifically, significant improvements for the CS condition compared to the PC were evidenced for sleep quality, sleep efficiency and HRQoL items of Mental Health, Feeling Healthy and Full of Energy, and Poor Physical or Mental Health preventing participants from doing their usual activities. Daytime fatigue was also significantly reduced from baseline for the CS condition, but the difference compared to the PC was less defined. Nonsignificant trends for the CS outperforming the PC were evidenced for stress/anxiety, several mood outcomes, and pain‐affected sleep.

We found that the CS formulation was well‐tolerated and resulted in improved sleep quality and efficiency. These sleep improvements in sleep quality are consistent with other studies on cannabinoids that found improved sleep quality and related health outcomes [32, 33]. Additionally, we found significant improvements in HRQoL outcomes and daytime fatigue measures, that correspond to the improved sleep quality.

These findings also support research that sleep quality is associated with improved daytime energy and productivity [34]. Sleep quality, a multidimensional construct integrating parameters such as sleep duration, sleep satisfaction, and sleep disruptions, is a key contributor to daytime feelings of both energy and fatigue.

The findings of this study contribute to the growing body of evidence regarding the effects of cannabinoids on sleep quality and related outcomes [15, 16, 17, 18]. In addressing previous research, this study offers a more nuanced understanding of discrepancies in the literature by directly comparing our results to specific findings from earlier studies. While prior research has presented mixed results, particularly regarding nonsleep‐related outcomes, our findings highlight the potential of cannabinoids to improve sleep quality, efficiency, and health‐related quality of life in adults with subthreshold insomnia. These improvements align with several previous studies demonstrating positive effects of cannabinoids on sleep but differ from studies reporting null results, which may be attributed to variations in study design, cannabinoid formulations, dosages, or participant populations. Furthermore, our analysis acknowledges the complex interplay between sleep and secondary outcomes, such as mood and anxiety, offering additional context to previously inconclusive findings.

One challenge in assessing the efficacy of multi‐component or botanical formulations of cannabinoids is in separating an observed effect from a placebo effect. Historically, few studies showing clinical effects attributable to cannabinoid containing formulations have been completed with precise, repeated doses of the cannabinoids. Recently, precise dose‐associated data are more common, but controlling for other factors in these studies is still complex. Many people with poor sleep quality also have symptoms of anxiety, depression, or chronic pain. Of importance, botanical or “whole plant” cannabis or hemp containing products can produce effects on mood, cognition, and perception, especially at higher doses. Many cannabinoid products, especially those that are inhaled, or which have a taste, are easy to identify by consumers. This study addressed these issues by using a relatively low dose soft gel formulation intended to produce little to no side effect and which has no easily identifiable taste or smell.

Although this study aimed to establish a clear difference between the CS formulation and the PC, a placebo effect was identified. Nonsignificant improvements for the CS compared to the PC were found for the POMS mood subscales of tension, anger, fatigue, depression, and vigor, as well as anxiety.

Sleep research using cannabinoid supplementation is in its infancy and thus further research, using a variety of research designs and outcomes, is critical to advancing our understanding of cannabinoids on sleep and related quality of life metrics. Future studies with larger sample size and longer assessment periods in both clinical and nonclinical sleep populations are encouraged so that more definitive conclusions can be made regarding the potential for this supplement to positively impact sleep and health [1, 2, 3, 4].

In summary, the CS resulted in significant improvements in validated measures of sleep quality, efficiency, and HRQoL when compared to PC as well as baseline. Improvements in daytime fatigue, pain affected sleep, mood, and anxiety were also associated with CS, though these were nonsignificant condition differences. In conclusion, Sanna Sleep supplementation may be a simple, effective, and well‐tolerated alternative to improve sleep quality and related health outcomes in adults with subthreshold insomnia symptoms.

## Author Contributions

**Heather Hausenblas:** conceptualization, investigation, funding acquisition, writing – original draft, methodology, validation, visualization, writing – review and editing, project administration, supervision, resources, software. **Stephanie Hooper:** investigation, methodology, validation, writing – review and editing, software, formal analysis, project administration, data curation, supervision. **Tarah Lynch:** methodology, project administration, data curation, supervision, investigation.

## Ethics Statement

This study received ethical approval from Sterling IRB. All participants signed an independent Institutional Review Board approved consent form (Sterling IRB, https://www.sterlingirb.com/).

## Conflicts of Interest

The authors declare no conflicts of interest.

## Transparency Statement

The lead author Heather Hausenblas affirms that this manuscript is an honest, accurate, and transparent account of the study being reported; that no important aspects of the study have been omitted; and that any discrepancies from the study as planned (and, if relevant, registered) have been explained.

## Data Availability

The data that support the findings of this study are available from the corresponding author upon reasonable request.
